# The complete mitochondrial genome of the cave shrimp *Typhlatya miravetensis* (Decapoda, Atyidae) and its systematic position 

**DOI:** 10.1080/23802359.2016.1238756

**Published:** 2016-11-12

**Authors:** José A. Jurado-Rivera, Damià Jaume, Carlos Juan, Joan Pons

**Affiliations:** a Department of Biology, Universitat de les Illes Balears, Palma de Mallorca, Spain;; b IMEDEA (CSIC-UIB) Mediterranean Institute for Advanced Studies, Esporles, Spain

**Keywords:** *Typhlatya*, mitogenome, phylogeny, decapoda, Atyidae

## Abstract

The complete mitochondrial genome of *Typhlatya miravetensis* from one of its only three known localities (Ullal de la Rabla de Miravet, Castellón, Spain) is presented here. The mitogenome is 15,865 bp in length and includes the standard set of two rRNAs, two non-coding regions plus 13 protein-coding genes. The later have been used to perform a phylogenetic analysis together with other Caridea representatives with mitogenome data in GenBank, inferring a close relationship with the Hawaiian volcano shirmp (*Halocaridina rubra*) within the family Atyidae.

*Typhlatya* Creaser, 1936 is an obligate subterranean aquatic genus of atyid shrimps that comprises 17 species, showing a punctuated distribution throughout coastal continental and insular ground-waters of the Mediterranean, north-central Atlantic and east Pacific (Botello et al. 2013). Such peculiar biology and distribution have classically drawn the attention of evolutionary biologists (e.g. Croizat et al. 1974; Banarescu 1990; Stock 1993; Hunter et al. 2008; Botello et al. 2013). Although a few of the studies are based on the analysis of DNA sequences, our knowledge about the genetics of this group of organisms is still scarce. Here we present the first mitogenome sequence for the genus *Typhlatya*. The *T. miravetensis* Sanz & Platvoet 1995 sample was collected in 2009 in Ullal de la Rambla de Miravet (Castellón, Spain; 40° 06’ 73’’N, 00° 03’ 60’’W). Voucher specimen has been deposited at the DNA and tissue collection of the Biodiversity, Systematic and Evolution group (Bio6Evo) of the University of the Balearic Islands with accession number 3151.

Total DNA was purified and the mitogenome was amplified in two large amplicons through long-range PCR as described in Pons et al. (2014). Amplicons were sequenced using a Junior NGS 454 platform. Genes were annotated with DOGMA (Wyman et al. 2004) and MITOS (Bernt et al. 2013) webservers. A complete mitogenome of 15,865 bp was obtained (ENA accession number LT608343).

The mitochondrial genome shows an A + T bias (A + T = 66.28%) and the gene content including 13 protein-coding genes (PCGs), 22 tRNAs and two ribosomal genes. An AT-rich (79.94%) non-coding region of 1042 bp was found between *rrnS* and *trnI* genes and might correspond to the origin of replication. Gene arrangement matches the ancestral putative pancrustacean gene order. Most genes are encoded in the positive strand excepting *nad1, nad4, nad4L, nad5, rrnL, rrnS* and the tRNAs *L1, C, F, H, P, Q, V* and *Y*. Start codons match the canonical ATA or ATG except for *cox1* (CCG), *nad5* (GTG), *nad6* (ATC), *atp8*, *nad1* and *nad2* (ATT). Similarly, stop codons are the canonical TAA or TAG except for *cox2* that showed a truncated codon stop (T).

Representative species from all Caridea genera with mitogenome sequences available in GenBank plus three Dendrobranchiata taxa as outgroup were used to assess the phylogenetic placement of *T. miravetensis*, which showed a close relationship with *Halocaridina rubra* Holthuis, 1963 within the family Atyidae (Figure 1).

**Figure 1.**
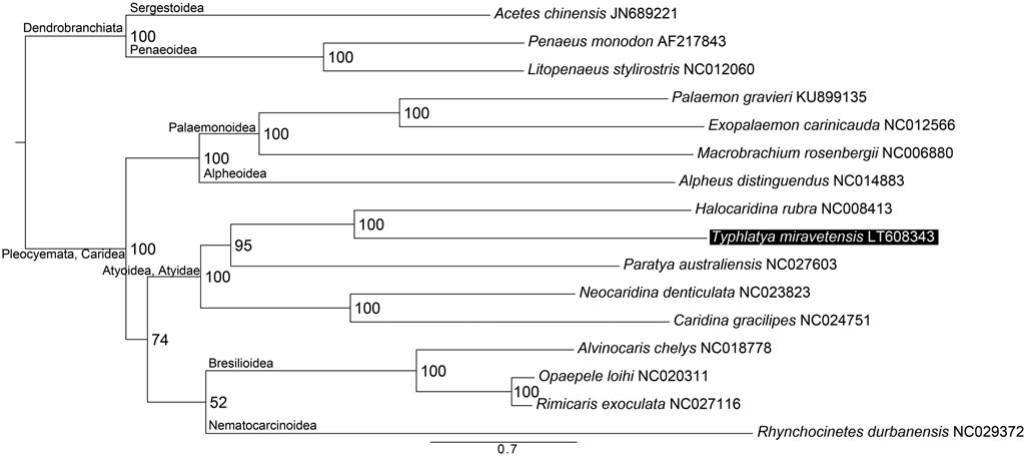
Maximum likelihood tree obtained using IQTREE v 1.3.12 (Nguyen et al. 2015) based on the PCGs of 12 Caridea species and three Dendrobranchiata outgroups highlighting the phylogenetic placement of *T. miravetensis*. DNA sequences were aligned in TranslatorX (Abascal et al. 2010). PartitionFinder v 1.1.1 (Lanfear et al. 2012) was used to select the best partitioning scheme and evolutionary models. Node numbers show bootstrap support values. GenBank accession numbers are given with species names.
